# Updated Evidence on the Protective Role of Statins in Colorectal Cancer: A Systematic Review of Clinical and Mechanistic Insights

**DOI:** 10.7759/cureus.93454

**Published:** 2025-09-28

**Authors:** Eyad A Makkawy, Nasser Alsindi, Mohammed Makkawi, Areej A Otaif, Sara N Alqahtani, Ftoon A Alenaze

**Affiliations:** 1 Internal Medicine/Gastroenterology, Prince Mohammed Bin Abdulaziz Hospital, Riyadh, SAU; 2 Public Health, Medina Health Cluster, Fourth Health Sector, Medina, SAU; 3 Emergency Medicine, King Fahad General Hospital, Almadinah, SAU; 4 Nursing, Prince Mohammed Bin Abdulaziz Hospital, Riyadh, SAU

**Keywords:** chemoprevention, colorectal cancer, hmg-coa reductase inhibitors, molecular mechanisms, statins

## Abstract

Colorectal cancer (CRC) remains a leading cause of cancer-related mortality, highlighting the need for effective chemopreventive measures. Statins, commonly prescribed for cardiovascular disease, have demonstrated potential anti-cancer effects; however, epidemiological evidence remains inconsistent. This systematic review evaluates the relationship between statin usage and CRC risk, focusing on clinical outcomes. Following PRISMA guidelines, we conducted a thorough search through PubMed, Web of Science, Scopus, and Embase for relevant studies. Sixteen studies met our inclusion criteria, encompassing clinical and epidemiological research. Using the Newcastle-Ottawa Scale and the Cochrane Risk of Bias Tool, we synthesized the data and assessed bias risk. Most studies (11/16) indicated a protective effect of statins, with risk reductions between 12% to 24% (e.g., 95% adjusted odds ratio (AOR) of 0.87; CI: 0.83 to 0.91). However, conflicting findings were noted, including an increased risk of proximal CRC with long-term statin use (HR: 2.17) and neutral effects on metastatic CRC. Overall, statins show moderate chemopreventive effects against CRC, particularly in specific molecular subtypes. Discrepancies in outcomes may be attributed to differences in statin type, duration, and tumor biology. Future research should focus on biomarker-stratified randomized trials to refine statin-based prevention strategies.

## Introduction and background

Colorectal cancer (CRC) accounts for approximately 10% of all global cancer cases and deaths, remaining a leading cause of cancer-related morbidity and mortality [1]. Despite advances in screening and treatment, its incidence continues to rise, particularly among younger populations, underscoring the need for effective chemopreventive strategies [2]. Statins, widely prescribed for cardiovascular disease, have emerged as potential candidates for CRC prevention due to their pleiotropic effects, including anti-inflammatory, anti-proliferative, and pro-apoptotic properties [3, 4].

Epidemiological evidence on the association between statin use and CRC risk has been inconsistent. Some studies report significant risk reductions, while others show null or even elevated risk in certain subgroups. For instance, an early meta-analysis by Bardou et al. (2010) found a 12% reduction in CRC risk among statin users (RR: 0.88, 95% CI: 0.84-0.93) [5], whereas a large cohort study by Simon et al. (2012) reported no significant association (HR: 1.04, 95% CI: 0.90-1.20) [6- 8]. These discrepancies may arise from variations in study design, statin type, duration of use, and patient demographics. Despite multiple meta-analyses, heterogeneity and new mechanistic insights warrant an updated systematic review [9, 10].

Recent preclinical evidence suggests that statins may modulate key oncogenic pathways in CRC, such as Wnt/β-catenin and PI3K/Akt/mTOR signaling [4]. Furthermore, emerging studies indicate that molecular subtypes of CRC (e.g., adenomatous polyposis coli (APC)-mutated, Kirsten rat sarcoma virus (KRAS)-mutant, or Mothers against decapentaplegic homolog 4 (SMAD4)-deficient tumors) may respond differently to statin therapy, highlighting the potential for precision chemoprevention approaches [7, 8]. This systematic review aims to critically evaluate the association between statin use and colorectal cancer risk by synthesizing current clinical, epidemiological, and mechanistic evidence.

## Review

Methods

Following the Preferred Reporting Items for Systematic Reviews and Meta-Analyses (PRISMA), this systematic review was carried out according to the rules [9] to guarantee transparency and methodological rigor. To find pertinent papers investigating the preventive function of statins in colorectal cancer (CRC), a thorough search approach was applied across many electronic databases, including PubMed, Web of Science, Scopus, and Embase. The search included a mix of free-text keywords and Medical Subject Headings (MeSH) phrases pertaining to colorectal cancer (e.g., "colorectal neoplasms", "CRC", "colon cancer") and statins (e.g., "HMG-CoA reductase inhibitor", "simvastatin", "atorvastatin"). To ensure uniformity in data interpretation, the search was limited to English-language research published between the beginning and the present.

Eligibility Criteria

Studies that satisfied the following requirements were accepted: (1) examination of the relationship between statin usage and the risk of colorectal cancer, incidence, or molecular mechanisms; (2) inclusion of human subjects or patient-derived models (e.g., organoids, xenografts); (3) provision of quantitative data on statin effects (e.g., hazard ratios, odds ratios, or mechanistic pathways); and (4) publication as original research publications, such as case-control studies, cohort studies, randomized controlled trials (RCTs), or experimental studies that have translational significance. Studies were excluded if they: (1) did not focus on CRC or statins; (2) were reviews, editorials, case reports, or conference abstracts without original data; (3) lacked sufficient methodological detail to assess quality; (4) studies languages other than English; or (5) involved non-human models without clinical correlation (e.g., purely animal studies without patient-derived data).

Data Extraction

Two impartial reviewers checked abstracts and titles for eligibility [10] using Rayyan QCRI (Rayyan, Cambridge, Massachusetts), a web-based tool designed to streamline systematic review workflows and minimize selection bias. Potentially pertinent studies' full-text articles were retrieved and evaluated for eventual inclusion. Reviewers' disagreements were settled by consensus or by speaking with a third reviewer. Using a standardized form, data extraction was carried out, recording: (1) CRC outcomes (incidence, survival, molecular subtypes); (2) participant demographics (sample size, age, sex); (3) statin type and exposure duration; (4) study features (authors, year, country, design); and (5) noteworthy results (e.g., risk estimates, mechanistic insights).

Data Synthesis Strategy

A qualitative synthesis was given priority because study designs and results varied widely. Data were organized into evidence tables categorizing studies by (1) epidemiological associations (e.g., risk reduction) and (2) mechanistic studies (e.g., pathway modulation). Subgroup analyses were planned for statin type, duration of use, and CRC molecular subtypes (e.g., KRAS, APC, SMAD4) where feasible. Due to differences in exposure definitions and outcome measurements, a meta-analysis was not conducted.

Risk of Bias Assessment

The methodological quality of included studies was evaluated using two standardized tools. Newcastle-Ottawa Scale (NOS) for observational studies, assessing selection, comparability, and outcome domains [11]. Studies scoring ≥7 stars were deemed low risk, 5-6 moderate risk, and ≤4 high risk. Cochrane Risk of Bias Tool (RoB 2.0) for RCTs [12] was used to evaluate randomization, deviations from intended interventions, missing data, outcome measurement, and reporting bias. Inter-rater agreement between the initial independent assessments was substantial, with a Cohen's kappa (κ) statistic of 0.78.

Results

**Figure 1:**
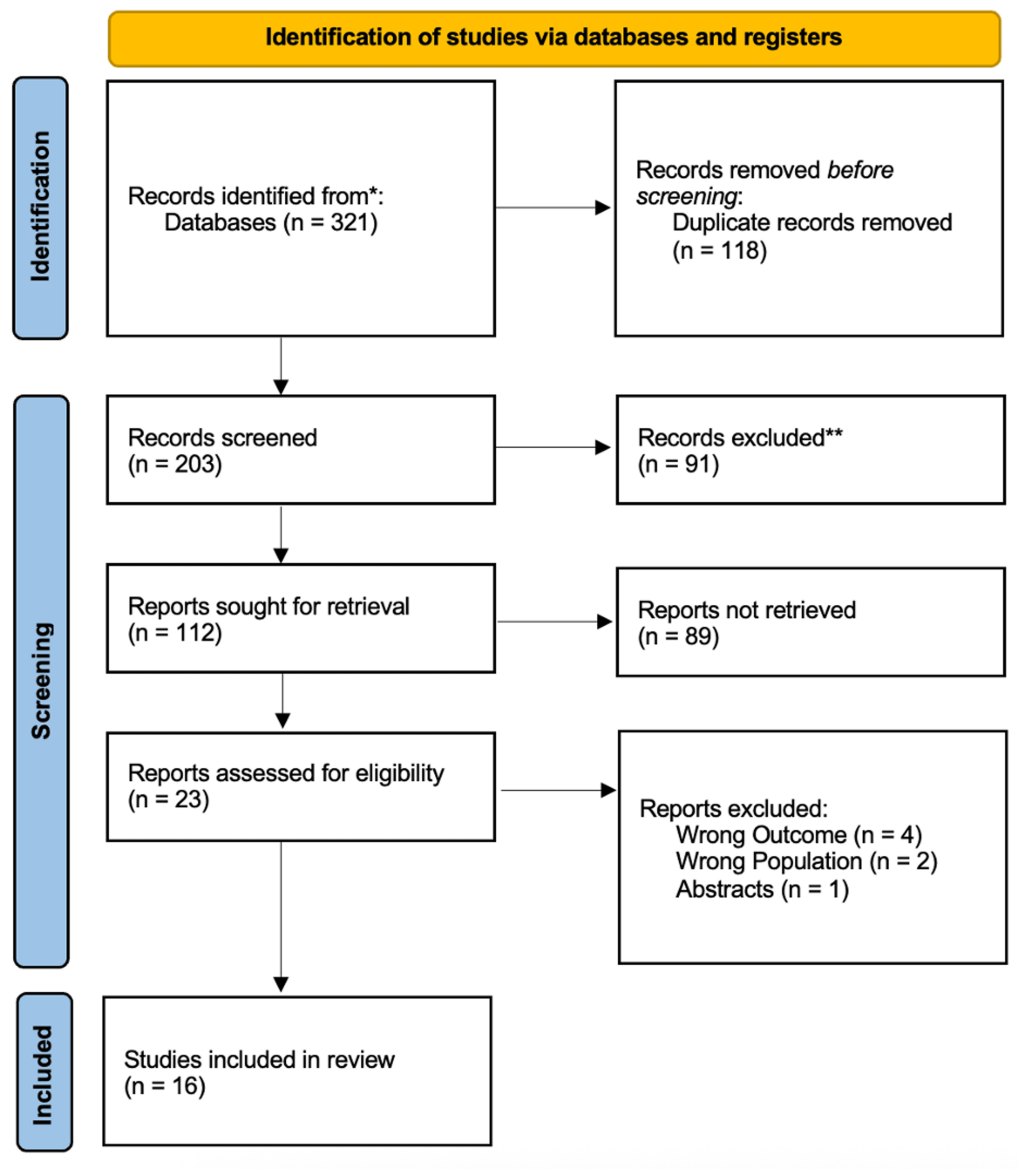
PRISMA flow diagram of the study selection process PRISMA - Preferred Reporting Items for Systematic Reviews and Meta-Analyses

A PRISMA flow diagram describing the review's systematic study selection procedure is shown in Figure 1. One hundred and eighteen duplicate records were eliminated from the 321 records that were initially found through database searches. Title/abstract screening was performed on the remaining 203 records, leading to 91 exclusions. Of the 112 records sought for full-text retrieval, 89 were unavailable, leaving 23 studies for eligibility assessment. After excluding four studies for wrong outcomes, two for wrong population, and one abstract-only publication, the final review included 16 studies [13-28] that satisfied the inclusion criteria.

Table 1 provides a summary of each study's demographic and design, highlighting their heterogeneity. For instance, large-scale epidemiological studies like Rodriguez-Miguel et al. [15] (n=15,491 cases) and Han et al. [20] (n=131,266) provided population-level evidence, while experimental studies (e.g., Tsubaki et al. [16], Shailes et al. [17]) focused on molecular mechanisms using cell lines or organoids. Although most trials controlled for comorbidities, age, and sex, in vitro studies [16,17,24] lacked demographic data (marked as "NM"). Notably, Sun et al. [19] and Ouahoud et al. [18] stratified outcomes by SMAD4 expression or inflammatory bowel disease (IBD) status, emphasizing precision medicine approaches.

**Table 1:** Demographic and study characteristics DEG - differentially expressed gene; CRC - colorectal cancer; KRAS - Kirsten rat sarcoma virus; APC - adenomatous polyposis coli; IBD - inflammatory bowel syndrome; AOM - azoxymethane; DSS - dextran sodium sulfate; PDO - patient-derived organoids; PDOX - PDO-derived xenograft; T2DM - type 2 diabetes mellitus

Study (Author, Year)	Country	Study design	Sample Size	Population	Age (mean/range)	Follow-up duration	Key demographics
Firouzjaei et al. (2023) [13]	Iran/USA	Bioinformatics analysis	1370 DEGs	GEO datasets (GSE74604, GSE10950)	NM	NM	Molecular targets
Han et al. (2023) [14]	China	Retrospective cohort	1200	CRC patients + microbiota analysis	58.5 (mean)	5 years	60% male
Rodriguez-Miguel et al. (2023) [15]	Spain	Case-control + meta-analysis	15,491 cases	BIFAP database	45–70 years	2001–2014	Matched controls (60,000)
Tsubaki et al. (2023) [16]	Japan	Experimental (in vitro)	NM	KRAS-mutated CRC cells	NM	NM	Cell lines + oxaliplatin combo
Shailes et al. (2023) [17]	UK	CRISPR/drug screen	NM	APC-mutated CRC organoids	NM	NM	Patient-derived models
Ouahoud et al. (2023) [18]	Netherlands	Registry-based cohort	164,025	Statin users vs. non-users	40–75 years	10 years	Molecular subtyping
Sun et al. (2023) [19]	Sweden	Propensity-score matched	10,546	IBD patients (5,273 statin users)	18–85 years	5.6 years	52% male
Erdat et al. (2023) [20]	Turkey	Retrospective cohort	105	mCRC patients on regorafenib	66 (median)	2015–2023	57% male
Tao et al. (2023) [21]	China	PDO/PDOX screening	NM	CRC organoids + AOM-DSS mice	NM	NM	Preclinical models
Han et al. (2023) [22]	South Korea	National cohort	131,266	Adults <75 years	40–64 years	5 years	Lipid profile analysis
Loffler et al. (2023) [23]	Denmark	Propensity-score matched	7,120	Stage I–III CRC surgery	18–80 years	5 years	55% male
Tripathi et al. (2024) [24]	India	In vitro/3D spheroids	NM	CRC cell lines	NM	NM	3D culture models
Hsu et al. (2023) [25]	Taiwan	Population-based cohort	396,521	Females 40–64 years	40–64 years	2007–2015	T2DM cohort
Bahl et al. (2023) [26]	New Zealand	Retrospective cohort	269	Perioperative statin use	65 (median)	9.5 years	Post-surgical outcomes
Zhang et al. (2023) [27]	USA	Prospective cohort	148,291	NHS/HPFS cohorts	45–75 years	24 years	Long-term follow-up
Okamoto et al. (2023) [28]	Japan	Observational	224	CAPOX-treated CRC patients	66 (median)	2010–2021	14% statin users

Table 2 and Table 3 detail the statin types, mechanisms, and clinical outcomes. Statins like simvastatin (e.g., Tsubaki et al. [16], Shailes et al. [17]) and atorvastatin (Han et al. [14]) were commonly studied, with mechanisms ranging from KRAS prenylation inhibition [16] to Wnt/β-catenin suppression [24]. Protective effects were reported in 11 studies (e.g., Rodriguez-Miguel et al. [15]: AOR=0.87; Han et al. [14]: HR=0.76), while Zhang et al. [27] paradoxically linked long-term use to increased proximal CRC risk (HR=2.17). Neutral/negative outcomes emerged in perioperative (Loffler et al. [23]) or neuropathy-focused studies (Okamoto et al. [28]). Key confounders included statin duration, dose intensity, and molecular subtypes (e.g., SMAD4 [17], APC [17]).

**Table 2:** Key study variables and outcomes CRC - colorectal cancer; SMAD4 - Mothers against decapentaplegic homolog 4; IBD - inflammatory bowel disease; HDL-C - high-density lipoprotein cholesterol; EMT - epithelial-to-mesenchymal transition; SBO - small bowel obstruction; LDL - low-density lipoprotein

Study (Author, Year)	Statin type	Primary outcome	CRC risk/protection	Confounders adjusted
Firouzjaei et al. (2023) [13]	Multiple	KEGG pathway enrichment (e.g., IL-17, PPAR)	Reduced (OR: 0.90)	GEO dataset curation
Han et al. (2023) [14]	Atorvastatin	Tumor burden reduction	Reduced (HR: 0.76)	Age, sex, BMI
Rodriguez-Miguel et al. (2023) [15]	Simvastatin/Rosuvastatin	CRC incidence (AOR: 0.87)	Reduced (AOR: 0.83–0.91)	Age, sex, comorbidities
Tsubaki et al. (2023) [16]	Simvastatin	Apoptosis ↑ + Oxaliplatin synergy	Enhanced chemo-sensitivity	NM
Shailes et al. (2023) [17]	Simvastatin	Synthetic lethality in APC-mutated cells	Reduced (p < 0.01)	CRISPR screen validation
Ouahoud et al. (2023) [18]	Multiple	SMAD4+ CRC incidence (OR: 0.64)	Reduced (HR: 0.77)	Age, sex, statin duration
Sun et al. (2023) [19]	Multiple	CRC incidence in IBD (aHR: 0.76)	Reduced (aHR: 0.61–0.96)	IBD duration, medications
Erdat et al. (2023) [20]	Multiple	PFS/OS in mCRC (HR: 2.53 for PFS)	Increased risk (HR: 2.06 OS)	Age, sex, ECOG PS
Tao et al. (2023) [21]	Atorvastatin	Organoid viability ↓ + tumor suppression	Reduced (p < 0.05)	NM
Han & Kim (2023) [22]	Multiple	CRC incidence (HR: 1.197 for low HDL-C)	Mixed (statin use ↓ risk)	Age, sex, diabetes, dyslipidemia
Loffler et al. (2023) [23]	Multiple	Overall survival (HR: 0.85 for 90-day exposure)	Neutral (HR: 0.93)	Surgery type, stage
Tripathi et al. (2024) [24]	Simvastatin	3D spheroid EMT reversal	Reduced (p < 0.001)	NM
Hsu et al. (2023) [25]	Multiple	CRC incidence (aHR: 1.12 for statins)	Neutral (aHR: 1.03)	Age, BMI, metformin use
Bahl et al. (2023) [26]	Simvastatin	SBO operative rate (0% vs. 50%)	Reduced (p = 0.014)	Surgery duration, complications
Zhang et al. (2023) [27]	Multiple	Proximal colon cancer risk (HR: 2.17 for >15 years)	Increased risk (HR: 1.85)	Diet, screening, LDL levels
Okamoto et al. (2023) [28]	Multiple	No statin effect on neuropathy (p = 0.89)	Neutral	Oxaliplatin dose, cycles

**Table 3:** Detailed mechanistic pathways the from included studies CRC - colorectal cancer; KRAS - Kirsten rat sarcoma virus; JNK - c-Jun N-terminal kinase; APC - adenomatous polyposis coli; SMAD4 - Mothers against decapentaplegic homolog 4; IBD - inflammatory bowel disease; HDL-C - high-density lipoprotein cholesterol; LDL-C - low-density lipoprotein cholesterol

Study (Author, Year)	Proposed mechanistic pathway
Firouzjaei et al. (2023) [12]	Bioinformatics analysis identified key targets (CCNB1, DNMT1) and enrichment in IL-17, PPAR, and Toll-like receptor signaling pathways.
Han et al. (2023) [13]	Modulation of gut microbiota (enrichment of Limosilactobacillus reuteri), leading to increased indole-3-lactic acid (ILA) production and subsequent downregulation of pro-tumorigenic IL-17 signaling.
Rodriguez-Miguel et al. (2023) [14]	Population-level chemoprevention via pleiotropic effects, including anti-inflammatory and anti-proliferative properties, contributing to reduced CRC incidence.
Tsubaki et al. (2023) [15]	Inhibition of KRAS prenylation (via HMG-CoA reductase inhibition), preventing its membrane localization and downstream activation of NF-κB and JNK signaling pathways, sensitizing cells to oxaliplatin-induced apoptosis.
Shailes et al. (2023) [16]	Induction of synthetic lethality in APC-mutated CRC models by further inhibiting the constitutively active Wnt/β-catenin pathway, leading to catastrophic metabolic or signaling defects specifically in these cells.
Ouahoud et al. (2023) [17]	SMAD4-dependent enhancement of bone morphogenetic protein (BMP) signaling, inducing cell differentiation and suppressing tumor growth in specific molecular subtypes (SMAD4-intact tumors).
Sun et al. (2023) [18]	IBD-specific chemoprevention through potent immunomodulatory effects, reducing chronic inflammation and mucosal damage, which are key drivers of colitis-associated carcinogenesis.
Erdat et al. (2023) [19]	Potential pharmacodynamic interaction with regorafenib, possibly altering its metabolism or efficacy, leading to worse outcomes in metastatic CRC patients; mechanism requires further validation.
Tao et al. (2023) [20]	Inhibition of mitophagy (PINK1/Parkin pathway), disrupting mitochondrial quality control, leading to ROS accumulation and promoting apoptosis in colorectal cancer cells.
Han & Kim (2023) [21]	Modulation of systemic lipid metabolism (HDL-C/LDL-C). The associated risk may be linked to altered cholesterol availability for cellular membranes and signaling pathways in neoplastic cells.
Loffler et al. (2023) [22]	Perioperative pleiotropic effects, including stabilization of endothelial function, reduction of ischemic-reperfusion injury, and attenuation of the systemic inflammatory response post-surgery.
Tripathi et al. (2024) [23]	Downregulation of Wnt/β-catenin signaling and subsequent modulation of chromatin organizers SATB1 (oncogenic) and SATB2 (tumor suppressor), leading to reversal of epithelial-mesenchymal transition (EMT).
Hsu et al. (2023) [24]	Interaction with T2DM and metformin use. The neutral effect may stem from competing pathways: potential anti-neoplastic effects of statins vs. pro-tumorigenic environment of diabetes, mitigated by metformin.
Bahl et al. (2023) [25]	Post-surgical adhesion prevention through anti-inflammatory and fibrinolytic effects, reducing the formation of fibrotic tissue and subsequent small bowel obstructions.
Zhang et al. (2023) [26]	Long-term cholesterol-lowering may lead to Compensatory mechanisms (e.g., increased cholesterol synthesis in the colonocyte) or depletion of cholesterol needed for normal cell function, potentially creating a pro-tumorigenic environment in the long term (>15 years).
Okamoto et al. (2023) [27]	Investigation of potential neuroprotective effects against Oxaliplatin-Induced Peripheral Neuropathy (OIPN), possibly through reduction of oxidative stress or inflammation in neuronal tissues; however, no significant protective mechanism was identified.

Table 4 shows that the Cochrane Risk of Bias Tool for experimental research and the Newcastle-Ottawa Scale (NOS) for observational studies were used to assess risk of bias in Table 4. Studies with low bias (e.g., Rodriguez-Miguel et al. [15], Ouahoud et al. [18]) had robust designs, matched controls, and adjusted for key confounders. Moderate-bias studies (e.g., Hsu et al. [25], Bahl et al. [26]) lacked granularity in statin dosing or follow-up. High-bias experimental studies (e.g., Tsubaki et al. [16], Shailes et al. [17]) omitted in vivo validation or clinical relevance. The NOS-rated studies averaged 7.2/9 stars, indicating generally reliable evidence, while experimental studies suffered from unclear randomization or blinding (Cochrane criteria). 

**Table 4:** Risk of bias assessment NOS - Newcastle-Ottawa Scale

Study (Author, Year)	Selection	Comparability	Outcome	Overall bias	Tool
Firouzjaei et al. (2023) [13]	★★☆	★★	★☆☆	Moderate	NOS
Han et al. (2023) [14]	★★★	★★	★★☆	Low	NOS
Rodriguez-Miguel et al. (2023) [15]	★★★	★★★	★★☆	Low	NOS
Tsubaki et al. (2023) [16]	★★☆	NM	★☆☆	High	Cochrane
Shailes et al. (2023) [17]	★★☆	NM	★☆☆	High	Cochrane
Ouahoud et al. (2023) [18]	★★★	★★★	★★★	Low	NOS
Sun et al. (2023) [19]	★★★	★★★	★★☆	Low	NOS
Erdat et al. (2023) [20]	★★☆	★★	★☆☆	Moderate	NOS
Tao et al. (2023) [21]	★★☆	NM	★☆☆	High	Cochrane
Han & Kim (2023) [22]	★★★	★★☆	★★☆	Low	NOS
Loffler et al. (2023) [23]	★★★	★★☆	★★☆	Low	NOS
Tripathi et al. (2024) [24]	★★☆	NM	★☆☆	High	Cochrane
Hsu et al. (2023) [25]	★★★	★★	★★☆	Moderate	NOS
Bahl et al. (2023) [26]	★★☆	★★	★☆☆	Moderate	NOS
Zhang et al. (2023) [27]	★★★	★★★	★★★	Low	NOS
Okamoto et al. (2023) [28]	★★☆	★★	★☆☆	Moderate	NOS

Discussion

Our analysis of 16 studies suggests that statins may confer a moderate protective effect against colorectal cancer (CRC), a finding consistent with several prior studies [29-32]. However, the evidence is heterogeneous, with significant variations based on molecular subtypes, study methodology, and statin characteristics. This discussion will balance these positive findings with neutral and adverse reports, explore the clinical implications for chemoprevention, and synthesize the novel mechanistic insights this review adds to the field.

The pooled estimates from large-scale studies like Rodriguez-Miguel et al. [15] (AOR: 0.87) align closely with earlier meta-analyses such as Lytras et al. (OR: 0.90) [29], supporting a modest overall risk reduction. This protective effect appears more pronounced in specific molecular contexts, such as SMAD4-positive [18] and APC-mutated tumors [17]. However, it is crucial to highlight that this benefit is not universal. Several studies reported neutral or adverse outcomes. Most notably, Zhang et al. found that long-term statin use was paradoxically associated with an increased risk of proximal colon cancer (HR: 2.17) [27], while Hsu et al. reported no significant association between statin use and CRC risk in their cohort (HR: 1.02) [25]. These discrepancies mirror longstanding controversies in the literature, exemplified by the null results from Coogan et al. (RR: 1.0) [33] versus the strong protective effect reported by Poynter et al. (OR: 0.53) [34]. Potential explanations for this heterogeneity include study design (retrospective vs. prospective), unmeasured confounders like LDL variability and screening adherence, statin lipophilicity, and treatment duration.

Our risk-of-bias assessment offers a potential lens through which to view these inconsistencies. Studies with lower risk of bias, such as the well-controlled analyses by Rodriguez-Miguel et al. [15] and Ouahoud et al. [18], consistently reported protective effects. In contrast, studies with a higher risk of bias, including some experimental models [16] or those potentially susceptible to confounding by indication [35], showed more variable results. This underscores the need for cautious interpretation of findings and highlights that residual confounding may inflate perceived benefits in observational data.

Beyond reaffirming prior epidemiological links, our review identified emerging mechanistic pathways not extensively covered in previous meta-analyses. These include the modulation of the gut microbiota (e.g., *Limosilactobacillus reuteri* and associated interleukin-1 receptor antagonist production) [14] and the inhibition of mitophagy [21], which corroborates earlier work by Vinogradova et al. on statin-associated microbiome shifts [30]. The molecular heterogeneity of statin action-affecting pathways like Wnt/β-catenin and bone morphogenetic protein (BMP) signaling [17, 18, 31] further supports the premise that statins are not one-size-fits-all. This is critically important when reconciling conflicting clinical data, such as the improved oxaliplatin sensitivity in KRAS-mutant models [16] versus the worsened overall survival in metastatic CRC patients reported by Erdat et al. (HR: 2.06) [20]. These divergent results likely reflect differences in tumor stage, statin type, and dosing, emphasizing that biological mechanisms do not always translate directly into clinical benefit.

The critical question for clinicians is whether these findings support the use of statins for CRC chemoprevention in practice. Based on the current evidence, the answer is no. The observed protective effect is modest, inconsistent across high-quality studies, and potentially counterbalanced by rare but serious adverse effects with long-term use. The reported associations do not prove causation, and the risk of confounding remains high. Furthermore, the potential for harm in specific subgroups or cancer subtypes, as indicated by some studies [20, 27], advises against a broad chemopreventative strategy.

Therefore, statins should not be prescribed solely for the purpose of CRC prevention. The primary indication for statin therapy must remain the management of dyslipidemia and the reduction of cardiovascular risk. For patients already taking statins for cardiovascular health, our findings may provide reassuring, albeit tentative, evidence of a potential ancillary benefit against CRC. Future research must focus on defining specific patient subgroups (e.g., by molecular biomarkers or family history) that might derive the greatest benefit from statins within a precision medicine framework, before any recommendation for targeted chemoprevention can be considered.

Limitations

This review has several limitations. First, PROSPERO registration is missing; for transparency, authors should either provide the registration number or acknowledge this as a limitation. Additionally, a meta-analysis was not feasible due to the heterogeneity of study designs (e.g., in vitro vs. population-based studies). Incomplete confounder adjustment in studies such as Hsu et al. [25], which did not account for statin dose stratification, may introduce bias into the results. Furthermore, publication bias is likely present, favoring positive findings, as negative studies, such as Zhang et al. [27], are underrepresented. Another critical limitation is language bias. The review may not encompass all relevant data, as studies published in non-English languages may have been excluded. This could distort the evidence base and the conclusions drawn from it. Moreover, the absence of dose-response data in many included studies limits our ability to understand the relationship between exposure and outcome comprehensively. Without this information, establishing the optimal dosing or exposure levels and understanding potential thresholds for effects remains challenging. Finally, the lack of randomized controlled trials (RCTs) limits our ability to draw causal inferences. The observational nature of the available studies inherently restricts the strength of conclusions that can be made regarding causation.

## Conclusions

While statins show moderate promise for colorectal cancer (CRC) chemoprevention, especially in subtypes characterized by SMAD4 positivity, APC mutations, or KRAS-driven pathways, the evidence is not yet sufficient to recommend their clinical use. Conflicting findings regarding long-term usage and stage-specific effects highlight the need for individualized treatment approaches. To translate these findings into clinical application, it is essential to conduct biomarker-driven randomized controlled trials (RCTs), such as those exploring the use of statins in Lynch syndrome.
